# Physician Perceptions of the Safety and Efficacy of GLP-1 Receptor Agonists: Underestimation of Cardiovascular Risk Reduction and Discrepancies with Clinical Evidence

**DOI:** 10.3390/jcdd12010019

**Published:** 2025-01-07

**Authors:** Srikanth Krishnan, Pratyaksh K. Srivastava, Jayram Attaluri, Rebecca Nayeri, Dhananjay Chatterjee, Jay Patel, Ali Nsair, Matthew Budoff, Arash Nayeri

**Affiliations:** 1 The Lundquist Institute at Harbor-UCLA, Torrance, CA 90502, USA; 2 Department of Medicine, Division of Cardiology, University of California Los Angeles, Los Angeles, CA 90095, USA; 3 Ahmanson-UCLA Cardiomyopathy Center, Los Angeles, CA 90095, USA; 4 Faculty of Life Sciences & Medicine, Kings College London, London SE1 9NH, UK; 5 Department of Medicine, Division of Hematology-Oncology, VA Greater Los Angeles Healthcare System, Los Angeles, CA 90073, USA; 6 David Geffen School of Medicine, University of California Los Angeles, Los Angeles, CA 90095, USA; 7 Cedars Sinai Medical Center, Los Angeles, CA 90048, USA

**Keywords:** GIP GLP-1 receptor agonist, metabolic syndrome, obesity, weight loss, semaglutide, tirzepatide

## Abstract

Glucagon-like peptide-1 receptor agonists (GLP-1 RAs) and glucose-dependent insulinotropic polypeptide/GLP-1 receptor agonists (GIP/GLP-1 RAs) are emerging as effective treatments for obesity and cardiometabolic disease. This study evaluated physician perceptions of the safety and efficacy of semaglutide and tirzepatide through a questionnaire administered to 165 attending physicians specializing in internal or family medicine, with 122 responses received. Physicians reported an average patient weight loss of 9.22%, significantly lower than the 14.9% and 18.5% reported in the STEP and SURMOUNT trials, respectively. Estimated side effect rates (32.62%) were markedly lower than trial-reported rates (89.7% and 80.5%), while estimated discontinuation rates (8.59%) exceeded trial data. Cardiovascular benefits were perceived by 48.4% of physicians in diabetic patients, consistent with random guessing, and by only 39.3% in nondiabetic patients, significantly below random guessing expectations. These results highlight discrepancies between physician perceptions and clinical evidence, suggesting gaps in understanding regarding these agents’ efficacy and safety profiles. Addressing these gaps could enhance physician knowledge, patient adherence, and clinical outcomes.

## 1. Introduction

The dual pandemics of obesity and cardiometabolic disease indicate a significant and growing global health crisis [1]. These conditions, which are associated with increased morbidity and mortality, place substantial economic strain on healthcare systems worldwide. Projections indicate that one in every two American adults will be obese by 2030, with severe obesity becoming the most prevalent BMI category among women, non-Hispanic black adults, and low-income adults [2].

Obesity’s pathophysiological complexity extends beyond traditional lifestyle factors such as diet and exercise to include polygenic predisposition, environmental influences, epigenetic changes, disrupted sleep patterns, gut microbiota dysbiosis, and chronic psychosocial stress. These factors can independently or synergistically increase the risk of developing obesity and associated comorbidities [3]. Of particular concern is obesity’s role as an independent risk factor for cardiovascular disease, contributing to most cases of excess mortality in obese individuals through cardiovascular-specific mechanisms [3,4]. Because sustained body weight control has been shown to improve the cardiometabolic risk profile, novel therapeutic strategies for obesity and cardiometabolic disease management are urgently needed [5].

Incretin-based therapies, such as glucagon-like peptide 1 receptor agonists (GLP-1 RAs) and dual glucose-dependent insulinotropic polypeptide/glucagon-like peptide 1 receptor agonists (GIP/GLP-1 RAs) have emerged as transformative treatments for managing obesity and cardiometabolic risk. These therapies have demonstrated pleiotropic effects extending beyond glucose-lowering, including significant reductions in body weight, improvements in lipid profiles, decreased blood pressure and enhanced endothelial function [6,7]. Additionally, GLP-1 RAs, such as liraglutide and semaglutide, directly impact atherogenosis by modulating lipid oxidation, reducing macrophage infiltration, and attenuating formation of foam cells in atherosclerotic plaques [8,9,10]. Furthermore, these agents have demonstrated reductions in major cardiovascular events, as validated in landmark trials [11,12,13].

Given this context, evaluating physicians’ understanding of the safety and efficacy profiles of GLP-1 and GIP/GLP-1 RAs is both timely and important. To address this, we conducted a survey aimed at assessing the current knowledge among medical professionals regarding these therapeutic agents.

## 2. Materials and Methods

From February to March 2024, a structured questionnaire was distributed via email to 165 attending physicians who had completed a residency in internal medicine or family medicine, including those with further subspecializations. The survey aimed to assess physicians’ usage and perceptions of the GLP-1 RA semaglutide and the GIP/GLP-1 RA tirzepatide. The survey comprised of sections on demographic information, frequency of prescribing these agents, perceived efficacy of these agents, and familiarity with their safety profiles. The survey questions are shown in Table 1. Of the 165 physicians contacted, 122 (74%) returned completed surveys, which were included in the analysis.

Semaglutide and tirzepatide were chosen because of their extensively studied safety and efficacy profiles in treating obesity and their significant combined market share in the United States among these classes of medications at the time of this study.

Statistical analysis was performed via IBM SPSS Statistics Version 27. Descriptive statistics were calculated for the study variables, with frequencies and percentages computed for categorical variables and means and standard deviations (SDs) calculated for continuous variables. One-sample *t* tests were conducted to compare the physicians’ survey responses with clinical trial data from the Semaglutide Treatment Effect in People with Obesity 1 (STEP 1, ClinicalTrials.gov Number: NCT03548935), the Study of Tirzepatide in Participants with Obesity or Overweight 1 (SURMOUNT-1, ClinicalTrials.gov Number NCT04184622), STEP-4 (ClinicalTrials.gov number NCT03548987), and SURMOUNT-4 (ClinicalTrials.gov number NCT04660643) trials. These tests were used to analyze metrics, including average weight loss, the rate of side effects, weight rebound, and the discontinuation rate at 72 weeks. The survey responses provided the reference values, means, and SDs for each metric, whereas the clinical trial data were obtained from published results, specifying the test values for each comparison. Continuous variables were compared via one-sample *t* tests.

In addition, one-sample proportion tests were performed to compare the proportion of physicians reporting cardiovascular protection in diabetic patients and that of physicians reporting cardiovascular risk reduction in nondiabetic patients against a 50% reference value simulating random guessing. For all the statistical tests, the level of significance was set at 0.05.

## 3. Results

### 3.1. Data Interpretation

#### 3.1.1. Descriptive Statistics

The sample consisted of 122 physicians with an average age of 41.6 years (SD = 8.50) and a nearly equal distribution of male (49.2%) and female (50.8%) participants. Among the included physicians, 45.1% reported prescribing semaglutide and tirzepatide. The estimated mean reported weight loss among patients was 9.22% (SD = 4.04), with 49.3% of patients achieving at least 5% weight loss (SD = 18.39) and 18.81% achieving at least 20% weight loss (SD = 13.74). The estimated percentage of patients experiencing any side effects was 32.62% (SD = 15.96), with specific side effects including nausea (18.36%, SD = 11.12) and diarrhea (7.50%, SD = 4.95). The mean discontinuation rate at 72 weeks was estimated at 8.59% (SD = 5.85), and the mean weight rebound was estimated at 29.26% (SD = 20.58) (Table 2).

#### 3.1.2. Comparison of Physician Perceptions with Actual Rates in Previous Clinical Trials

One-sample *t* tests were performed to compare average weight loss, the rate of any side effects (%), weight rebound, and the discontinuation rate at 72 weeks.

#### 3.1.3. Average Weight Loss

The physician perception data revealed an average weight loss of 9.22% (SD = 4.038). This percentage was significantly lower than the weight loss reported in the STEP 1 trial (14.9%) and the SURMOUNT-1 trial (18.5%). One-sample *t* tests revealed statistically significant differences in the rates between the present study and previous studies: t(121) = −15.533, *p* < 0.001, with a mean difference of −5.679% (95% CI [−6.40, −4.95]) for the STEP 1 trial, and t(121) = −25.380, *p* < 0.001, with a mean difference of −9.279% (95% CI [−10.00, −8.55]) for the SURMOUNT-1 trial (Table 3).

#### 3.1.4. Side Effects (%)

**Figure 1:**
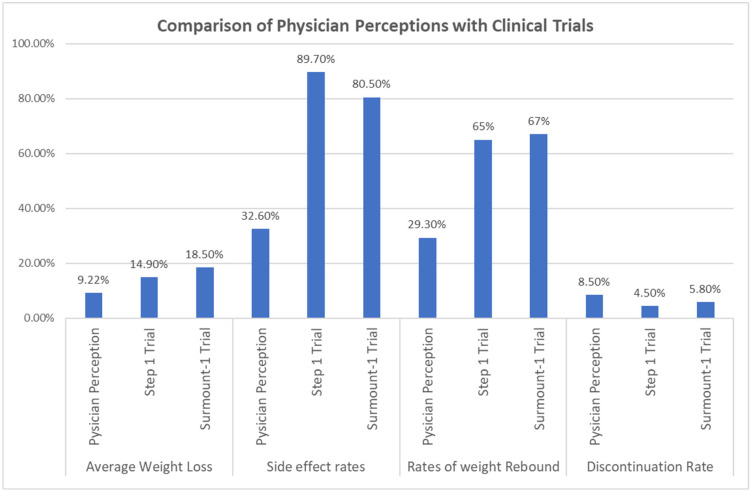
Comparison of Physician Perceptions with Clinical Trials.

The side effect rate estimated by the physicians was 32.62% (SD = 15.957). This proportion was significantly lower than the rates of side effects reported in the STEP 1 trial (89.7%) and the SURMOUNT-1 trial (80.5%) (Figure 1). The *t* tests revealed significant differences in the rate between the present study and previous studies: t(121) = −39.508, *p* < 0.001, with a mean difference of −57.077% (95% CI [−59.94, −54.22]) for the STEP 1 trial, and t(121) = −33.140, *p* < 0.001, with a mean difference of −47.877% (95% CI [−50.74, −45.02]) for the SURMOUNT-1 trial (Table 3).

#### 3.1.5. Weight Rebound

The mean weight rebound estimated by physicians was 29.26% (SD = 20.577), which was significantly lower than the weight rebound reported in the STEP 4 trial (65%) and the SURMOUNT-4 trial (67%) (Figure 1). The *t* tests indicated significant differences in the mean weight rebound between the present study and previous studies: t(121) = −19.183, *p* < 0.001, with a mean difference of −35.738% (95% CI [−39.43, −32.05]) for the STEP 4 trial, and t(121) = −20.257, *p* < 0.001, with a mean difference of −37.738% (95% CI [−41.43, −34.05]) for the SURMOUNT-4 trial (Table 3).

#### 3.1.6. Discontinuation Rate at 72 Weeks

The discontinuation rate at 72 weeks, as estimated by physicians, was 8.59% (SD = 5.855), which was significantly higher than that reported in the STEP-1 trial (4.5%) and the SURMOUNT-1 trial (5.8%) (Figure 1). The *t* tests indicated differences in the rate between the present study and previous studies: t(120) = 7.678, *p* < 0.001, with a mean difference of 4.087% (95% CI [3.03, 5.14]) for the STEP 1 trial,, and t(120) = 5.236, *p* < 0.001, with a mean difference of 2.787% (95% CI [1.73, 3.84]) for the SURMOUNT-1 trial (Table 3).

#### 3.1.7. Cardiovascular Protection and Risk Reduction

To compare cardiovascular protection in diabetic patients and cardiovascular reduction in nondiabetic patients, one-sample proportion tests were performed, and the results were compared with the 50% reference value simulating random guessing. For cardiovascular protection in diabetic patients, 59 out of 122 physicians (48.4%) reported a positive effect. This proportion was not significantly different from the 50% reference value (Z = −0.362, *p* = 0.359 one-sided, 95% CI: 0.397–0.571) (Table 3). For cardiovascular risk reduction in nondiabetic patients, 48 out of 122 physicians (39.3%) reported a positive effect. This proportion was significantly lower than the 50% reference value (Z = −2.354, *p* = 0.009 one-sided, 95% CI: 0.311–0.482) (Table 4).

## 4. Discussion

This survey provides insights into the perceptions of attending physicians on the safety and efficacy of the GLP-1 RA semaglutide and the GIP/GLP-1 RA tirzepatide. These medications are increasingly utilized in clinical practice, and our findings revealed statistically significant differences between physician perceptions and published clinical trial data across the examined metrics. Specifically, physicians underestimated the efficacy in terms of weight loss as well as the weight rebound following discontinuation. They also underestimated the rate of side effects while overestimating the discontinuation rate. Moreover, there is limited awareness of the broader cardiovascular benefits of these medications, particularly for nondiabetic patients.

These discrepancies represent an opportunity for ongoing education on the evolving role of diabetes medications in the treatment of obesity and cardiovascular disease. Historical challenges in demonstrating macrovascular benefits despite successful microvascular outcomes, including a paradoxical increase in the risk of cardiovascular and all-cause mortality caused by some diabetes therapies, led to a 2008 Food and Drug Administration (FDA) directive requiring cardiovascular safety evaluations for new diabetes treatments [14]. This directive facilitated large outcome trials confirming the class effect of sodium-glucose cotransporter-2 (SGLT-2) inhibitors in reducing major adverse cardiovascular events as well as cardiorenal benefits, including reductions in heart failure hospitalizations and progression to end-stage renal disease in both diabetic and nondiabetic patients [15,16]. These findings have led to a major shift in treatment guidelines [17].

Similarly, GLP-1 RAs have been shown to significantly reduce poor cardiovascular and renal outcomes among diabetic patients with established cardiovascular disease, as demonstrated in the Semaglutide Unabated Sustainability in Treatment of Type 2 Diabetes-6 (SUSTAIN-6, ClinicalTrials.gov number NCT01720446) and Liraglutide Effect and Action in Diabetes: Evaluation of Cardiovascular Outcome Results (LEADER, ClinicalTrials.gov number NCT01179048) trials [11,12].

In the SUSTAIN-6 trial, which included 3297 adults with type 2 diabetes and high cardiovascular risk, 60% of participants had a history of ischemic heart disease, 32% had a history of myocardial infarction, 11% had a history of ischemic stroke, and 22% had a history of heart failure. Treatment with semaglutide resulted in a 26% reduction in the composite primary endpoint of cardiovascular death, nonfatal stroke, and nonfatal myocardial infarction (hazard ratio (HR) 0.74, 95% CI 0.58–0.95, *p* = 0.02) [11].

In the LEADER trial, which included 9340 patients with type 2 diabetes at high cardiovascular risk, liraglutide significantly reduced the primary composite endpoint of major adverse cardiac events by 13% (HR 0.87, 95% CI 0.78–0.97, *p* = 0.01). This included a 22% reduction in the number of cardiovascular deaths (HR 0.78, 95% CI 0.66–0.93, *p* = 0.007) and a 15% reduction in all-cause mortality (HR 0.85, 95% CI 0.74–0.97, *p* = 0.02). The trial also reported a reduction in the progression of nephropathy (5.7% vs. 7.2%, HR 0.78, 95% CI 0.67–0.92, *p* = 0.003) [12].

Subsequent trials have shown the benefit of GLP-1 RAs in nondiabetic populations. The Semaglutide Effects on Cardiovascular Outcomes in People With Overweight or Obesity (SELECT, ClinicalTrials.gov Number: NCT03574597) trial demonstrated improved cardiovascular outcomes in overweight or obese nondiabetic patients with preexisting cardiovascular disease, with a 20% reduction in the number of major adverse cardiovascular events, which included death from cardiovascular causes, nonfatal myocardial infarction, or nonfatal stroke (HR 0.8, 95% CI 0.72–0.90, *p* < 0.0001) [13]. Additionally, the Semaglutide Treatment Effect in People With Heart Failure With Preserved Ejection Fraction (STEP-HFpEF, ClinicalTrials.gov Number: NCT04788511) trial demonstrated improvements in heart failure-related symptoms and physical limitations in obese diabetic patients with heart failure with preserved ejection fraction [18]. However, adverse effects were common, with gastrointestinal issues being the most frequently reported, at 74.2% with semaglutide compared to 47.9% for placebo. These issues included nausea, diarrhea, vomiting, and constipation [19].

Because of the cardiovascular and renal benefits to overweight and obese nondiabetic individuals, the FDA approved liraglutide for weight management based on the results of the Satiety and Clinical Adiposity—Liraglutide Evidence in Individuals With and Without Diabetes (SCALE, ClinicalTrials.gov Number: NCT01272219) trial [20]. Similarly, semaglutide has shown significant benefits in terms of weight loss in addition to cardiovascular and renal benefits. In the STEP-1 trial, semaglutide resulted in an average 14.9% reduction in body weight compared with 2.4% with placebo. Subsequent STEP trials confirmed significant weight loss with semaglutide compared with both placebo and liraglutide in both diabetic and nondiabetic patients [19].

Tirzepatide has similarly shown promising results in clinical trials. A series of trials titled “A Study of Tirzepatide in Participants With Type 2 Diabetes” (SURPASS-1 through SURPASS-5), investigating the efficacy and safety of tirzepatide as monotherapy or add-on therapy in managing type 2 diabetes. The SURPASS trials have shown significant weight loss and improvements in cardiovascular outcomes among participants [21]. Like semaglutide, tirzepatide is associated with a high incidence of gastrointestinal adverse effects; however, despite the high incidence of adverse effects, the discontinuation rate remains low for tirzepatide (5.8% in SURMOUNT-1) [22].

Ongoing trials aim to establish the safety and efficacy profile of tirzepatide for weight management and its potential impact on cardiovascular outcomes across various patient populations. The Study of Tirzepatide Compared With Dulaglutide on Major Cardiovascular Events in Participants With Type 2 Diabetes (SURPASS-CVOT, ClinicalTrials.gov number NCT04255433) trial is a large-scale cardiovascular outcomes trial comparing tirzepatide to dulaglutide in 13,299 patients with type 2 diabetes and established atherosclerotic cardiovascular disease [23].

With the increasing number of indications and the development of new drugs within this class, interest in these treatments among patients and healthcare providers is increasing. Our survey identified gaps in physician knowledge that could impact patient management. Specifically, the underestimation of side effects and the overestimation of discontinuation rates might influence patient counseling and expectations, potentially affecting treatment adherence and satisfaction. Moreover, a limited awareness of the cardiovascular benefits could lead to the underutilization of these treatments in eligible patients. Additionally, overestimation of long-term efficacy could set unrealistic expectations about the sustainability of weight loss with these therapies.

To bridge these gaps, we advocate for targeted, continuous education programs that focus on the comprehensive safety and efficacy profiles of these novel agents. Increased education and awareness may help physicians set more accurate expectations for patients, potentially improving adherence, satisfaction, and overall patient outcomes.

This study should be interpreted in the context of its limitations. The survey respondents were not chosen at random, and their understanding may not represent the average physician’s understanding of these agents. While efforts were made to include only those physicians likely to be familiar with these agents (internal medicine and family medicine specialists, excluding specialties less likely to prescribe these drugs), we did not collect data on subspecialization or years out of training. This decision was influenced by the desire to keep the survey brief and encourage maximal participation physicians who were not receiving honoraria. As a result, a broad range of demographic features were not captured. However, even if collected, the sample size would have limited statistical power to analyze these factors as predictors of understanding regarding the efficacy and safety of these drugs.

Questions regarding safety and efficacy did not distinguish between semaglutide and tirzepatide, as the survey aimed to assess general familiarity with incretin-based therapies rather than to directly compare individual agents. While this may have introduced some ambiguity, the reported estimates of safety and efficacy were markedly different from those published for either agent, and we believe this approach did not substantially alter the study’s findings. Additionally, we did not account for specific doses, despite the known differences in efficacy between varying dosages. Similarly, variations in weight loss at specific time points, such as the 72-week mark reported in seminal studies, were not addressed. Despite these limitations, the key finding of this study is that physician perceptions of weight loss were lower than the results demonstrated in the landmark trials for these drugs.

Finally, this study was designed as a thought-provoking assessment of physicians’ current understanding of these therapies at a specific point in time. Given the ongoing publication of new studies and large advertising campaigns, it is likely that physician knowledge and perceptions will evolve in the coming years.

## 5. Conclusions

Our survey revealed significant gaps in physician knowledge regarding the safety and efficacy of semaglutide and tirzepatide. These discrepancies between physician perceptions and clinical trial data demonstrate the need for targeted educational programs. The implementation of such programs may improve patient outcomes, patient satisfaction, and patient adherence to these treatments.

## Figures and Tables

**Table 1:** Medical professionals’ survey on the effectiveness and tolerability of weight loss medications.

Thank you for your participation in our survey of medical professionals’ perceptions and experiences with novel weight loss medications. Your anonymous responses will contribute to a broader understanding of how these weight loss treatments may be used in clinical practice.
Please answer the following questions based on your estimation or clinical experience:
Have you ever prescribed any of the following medications: semaglutide (Ozempic, Wegovy), tirzepatide (Mounjaro, Zepbound)?
In your estimation, what is the average percentage of weight loss in patients taking these weight loss medications?
In your estimation, what percentage of patients achieve at least 5% weight loss while on these medications?
In your estimation, what percentage of patients achieve at least 20% weight loss while on these medications?
Do you believe these medications provide secondary cardiovascular prevention benefits in overweight diabetic patients with established cardiovascular disease?
Do you believe these medications provide secondary cardiovascular prevention benefits in overweight non-diabetic patients with established cardiovascular disease?
In your estimation, what percentage of patients experience any side effects from these weight loss medications?
Please estimate the percentage of patients that experience nausea:
Please estimate the percentage of patients that experience diarrhea:
What is your estimation of the rate at which patients discontinue the use of weight loss medications due to any reason?
In patients who interrupt or stop using weight loss medication, what is your estimation of the percentage of weight that is regained?

**Table 2:** Descriptive statistics.

Characteristics	N (%)
Sex	
Male	60 (49.2)
Female	62 (50.8)
Age (years), Mean	41.6
Have you prescribed these agents?	
No	67 (54.9)
Yes	55 (45.1)
* CV Protection in diabetics	
No	63 (51.6)
Yes	59 (48.4)
CV reduction in non-diabetics	
No	74 (60.7)
Yes	48 (39.3)
Average weight loss (%), Mean ± SD	9.22 ± 4.04
At least 5% weight loss (%), Mean ± SD	49.30 ± 18.39
At least 20% weight loss (%), Mean ± SD	18.81 ± 13.74
Any side effect (%), Mean ± SD	32.62 ± 15.96
Nausea (%), Mean ± SD	18.36 ± 11.12
Diarrhea (%), Mean ± SD	7.50 ± 4.95
D/C rate at 72 weeks (%), Mean ± SD	8.59 ± 5.85
Weight rebound (%), Mean ± SD	29.26 ± 20.58

* CV—Cardiovascular.

**Table 3:** Comparison of physician perceptions with clinical trials.

Comparison	Physician Mean % (SD, n)	Reference Mean % (SD, n)	t-Value	*p*-Value	Mean Difference (95% CI)
Average weight loss					
Physicians’ vs. STEP 1 trial *	−9.22 (4.038, 122)	−14.9 (NR, 1306)	−15.533	<0.001	−5.679 (−6.40–−4.95)
Physicians’ vs. SURMOUNT-1 trial *	−9.22 (4.038, 122)	−18.5 (NR, 1896)	−25.380	<0.001	−9.279 (−10.00–−8.55)
Rate of side effects					
Physicians’ vs. STEP 1 trial *	32.62 (15.957, 122)	89.7 (15.95, 1306)	−39.508	<0.001	−57.077 (−59.94–−54.22)
Physicians’ vs. SURMOUNT-1 trial *	32.62 (15.957, 122)	80.5 (15.95, 1896)	−33.140	<0.001	−47.877 (−50.74–−45.02)
% Weight regained					
Physicians’ vs. STEP 4 trial *	29.26 (20.577, 122)	65 (15, 268)	−19.183	<0.001	−35.738 (−39.43–−32.05)
Physicians’ vs. SURMOUNT-4 trial *	29.26 (20.577, 122)	67 (15, 335)	−20.257	<0.001	−37.738 (−41.43–−34.05)
Discontinuation rate					
Physicians’ vs. STEP 1 trial *	8.59 (5.855, 121)	4.5 (NR, 1306)	7.678	<0.001	4.087 (3.03–5.14)
Physicians’ vs. SURMOUNT-1 trial *	8.59 (5.855, 121)	5.8 (6.2, 1896)	5.236	<0.001	2.787 (1.73–3.84)

Notes: * Significant at <0.01 level.

**Table 4:** Comparison of physician perceptions with reference value (50%).

Comparison	Observed Successes/Trials	Proportion (95% CI)	Test Value	Z Score	One-Tailed *p*-Value
CV protection in diabetics	59/122	0.484 (0.397–0.571)	0.5	−0.362	0.359
CV reduction in non-diabetics *	48/122	0.393 (0.311–0.482)	0.5	−2.354	0.009

Notes: * Significant at <0.01 level.

## Data Availability

Data are contained within the article and Supplementary Materials.

## Supplementary Materials

The following supporting information can be downloaded at: https://www.mdpi.com/article/10.3390/jcdd12010019/s1, Physician survey responses and trial raw data.
